# Riboflavin Has Neuroprotective Potential: Focus on Parkinson’s Disease and Migraine

**DOI:** 10.3389/fneur.2017.00333

**Published:** 2017-07-20

**Authors:** Eyad T. Marashly, Saeed A. Bohlega

**Affiliations:** ^1^Department of Neurosciences, King Faisal Specialist Hospital and Research Centre, Riyadh, Saudi Arabia

**Keywords:** riboflavin, Parkinson’s disease, migraine, oxidative stress, glutamate excitotoxicity, pyridoxal phosphate, homocysteine, kynurenine

## Abstract

With the huge negative impact of neurological disorders on patient’s life and society resources, the discovery of neuroprotective agents is critical and cost-effective. Neuroprotective agents can prevent and/or modify the course of neurological disorders. Despite being underestimated, riboflavin offers neuroprotective mechanisms. Significant pathogenesis-related mechanisms are shared by, but not restricted to, Parkinson’s disease (PD) and migraine headache. Those pathogenesis-related mechanisms can be tackled through riboflavin proposed neuroprotective mechanisms. In fact, it has been found that riboflavin ameliorates oxidative stress, mitochondrial dysfunction, neuroinflammation, and glutamate excitotoxicity; all of which take part in the pathogenesis of PD, migraine headache, and other neurological disorders. In addition, riboflavin-dependent enzymes have essential roles in pyridoxine activation, tryptophan-kynurenine pathway, and homocysteine metabolism. Indeed, pyridoxal phosphate, the active form of pyridoxine, has been found to have independent neuroprotective potential. Also, the produced kynurenines influence glutamate receptors and its consequent excitotoxicity. In addition, methylenetetrahydrofolate reductase requires riboflavin to ensure normal folate cycle influencing the methylation cycle and consequently homocysteine levels which have its own negative neurovascular consequences if accumulated. In conclusion, riboflavin is a potential neuroprotective agent affecting a wide range of neurological disorders exemplified by PD, a disorder of neurodegeneration, and migraine headache, a disorder of pain. In this article, we will emphasize the role of riboflavin in neuroprotection elaborating on its proposed neuroprotective mechanisms in opposite to the pathogenesis-related mechanisms involved in two common neurological disorders, PD and migraine headache, as well as, we encourage the clinical evaluation of riboflavin in PD and migraine headache patients in the future.

## Introduction

With the huge burden of neurological diseases on patient’s life and society resources, the need of finding and having neuroprotective agents is critical and cost-effective. In fact, the advances in medical research have found up to date multiple agents having unique proposed neuroprotective mechanisms and influencing different neurologic disease processes. Riboflavin is one of those proposed neuroprotective agents; however, its neuroprotective abilities have been underestimated in comparison to other known neuroprotective agents. Our focus in this article is to shed light on riboflavin neuroprotective characteristics, encouraging more research to be done in the future in this regard.

Riboflavin, a water-soluble vitamin, is part of the B complex vitamins, known as vitamin B-2. It is characterized by its unique bright yellow coloration of urine when taken in large amounts. Riboflavin plays a role in a wide range of metabolic pathways and processes, serving as a coenzyme for a variety of flavoprotein enzyme reactions. Riboflavin active forms are flavin mononucleotide (FMN) and flavin adenine dinucleotide (FAD).

Importantly, 10–15% of global population have an inherited condition of limited riboflavin absorption and utilization; leading to a potential biochemical riboflavin deficiency worldwide (1). In fact, based on erythrocyte glutathione reductase activation coefficient test (EGRAC), 54% of British non-elderly adult population was at least having borderline riboflavin deficiency (1). Indeed, riboflavin deficiency across European countries ranges between 7 and 20% (2).

As a matter of fact, neural tissue has a higher susceptibility to oxidative stress. Oxidative stress, a term refers to the injurious results in living organisms due to an imbalance favoring oxidants over antioxidants (3), has been implicated in multiple disease processes and aging. Oxidants are the normal results of *in vivo* interactions between oxygen and organic molecules. Concerning the brain, it forms 2% of total body weight with high levels of fatty acids, uses 20% of total body oxygen, and has lower antioxidant activity than other tissues. This gives the neural tissue a higher susceptibility to peroxidation (4) and oxidative damage in comparison to other tissues. In fact, oxidative stress has been implicated in multiple neurodegenerative disorder pathogenesis (4).

## Parkinson’s Disease (PD) Pathogenesis: Role of Oxidative Stress, Mitochondrial Dysfunction, and Neuroinflammation

Parkinson’s disease is a chronic, progressive neurodegenerative disorder involving the dopaminergic neurons in the substantia nigra pars compacta of the brain (5). To elaborate, increased levels of oxidized lipids (6), oxidized proteins (7), and oxidized DNA (7) and decreased levels of reduced glutathione (8) have been demonstrated in PD substantia nigra. In addition, substantia nigra dopaminergic neurons contain oxidant-generating enzymes, such as tyrosine hydroxylase and monoamine oxidase, as well as iron catalyzing the Fenton reaction producing superoxide and hydrogen peroxide radicals (9). Collectively, it is indicated that oxidative stress is a hallmark in the degenerative process of PD. The proposed elements that potentially cause oxidative stress in PD are dopamine metabolism, mitochondrial dysfunction, and neuroinflammation (5).

### Dopamine Metabolism

The neurotransmitter dopamine itself can be a source of oxidative stress. Oxidation of dopamine and consequent quinone modification contribute to the vulnerability of dopaminergic neurons (9). As a matter of fact, dopamine quinone species can modify cysteinyl residues and sulfhydryls, such as reduced glutathione, normally involved in neuronal survival (9). In addition, dopamine quinone species can dysfunctionally modify proteins involved PD pathophysiology, such as α-synuclein, parkin, DJ-1, and UCH-L1 (9). To add, dopamine quinone contributes to mitochondrial dysfunction (10) targeting Complex I and Complex III of electron transport chain, also, inactivates dopamine transporter and tyrosine hydroxylase (11). Eventually, dopamine quinone species can cyclize to become the highly reactive aminochrome (9), generating superoxide, depleting cellular NADPH, and ultimately forming neuromelanin (9), the final product of dopamine oxidation accumulated in the nigral region of the brain, which can trigger neuroinflammation exacerbating neurodegeneration.

### Mitochondrial Dysfunction

Neuronal ATP formation depends on mitochondrial aerobic respiration, which normally produces hydrogen peroxide and superoxide radicals as byproducts during mitochondrial oxidative phosphorylation (9). Mitochondrial dysfunction can cause a dramatic increase in reactive oxidant species (ROS) overwhelming the cellular antioxidant mechanisms. Environmental factors, such as neurotoxins, pesticides, insecticides, dopamine metabolism, and genetic mutations in PD-associated proteins contribute to mitochondrial dysfunction (5). Indeed, α-synuclein seems to inhibit mitochondrial Complex I (9), and dopamine quinone species target Complex I and Complex III of electron transport chain (10). The increase in ROS production is proportional to the degree of complex I inhibition (12). Subsequent to mitochondrial complex I inhibition, aconitase, a mitochondrial enzyme, is inactivated due to oxidation of its iron-sulfur clusters, in addition to the increased peroxidation of the mitochondrial phospholipid cardiolipin releasing cytochrome *c*, and eventually triggering apoptosis (13). Collectively, mitochondrial dysfunction leads to increased mitochondrial ROS contributing to PD pathogenesis.

### Neuroinflammation

In response to neural tissue injury or toxic insult, microglial cells undergo activation as a self-defensive mechanism. Upon activation, free radicals such as nitric oxide (NO) and superoxide are released, which elevates oxidative stress contributing to pathogen elimination and local tissue damage (9). Over-activation and/or chronic activation of microglia cause excessive and uncontrolled neuroinflammatory responses, leading to a self-perpetuating vicious cycle of neurodegeneration (14). Regarding PD, a greater density of activated microglia has been found in substantia nigra and olfactory bulb of both sporadic and familial PD patients (5). In fact, neurodegeneration in PD is associated with chronic neuroinflammation controlled essentially by activated microglia (9). To elaborate, PD-associated proteins like parkin, LRRK2, and DJ-1 have been reported to activate microglia (5), as well as molecules released by damaged dopaminergic neurons such as neuromelanin, α- synuclein, and active form of matrix metalloproteinase 3 (MMP-3) (9).

## Migraine Pathogenesis: Role of Oxidative Stress, Neuroinflammation, and Mitochondrial Dysfunction

Migraine is defined as a neurovascular disorder involving cortical spreading depression (CSD), neurogenic inflammation, and dysfunction in cranial vascular contractility (15).

### Oxidative Stress

Oxidative stress role in migraine pathogenesis is emphasized by multiple studies. In Alp et al. study (16), the levels of total antioxidants were decreased and the levels of total oxidants and the oxidative stress index were increased in patients with migraine without aura in comparison to controls, indicating an exposure to potent oxidative stress in migraine. In addition, in Tuncel et al. study (17), the malondialdehyde (MDA) levels of migraine patients were significantly higher than that in the controls. MDA reflects lipid peroxidation. In fact, elevated oxidative stress causes elevation in MDA (18). Consequently, migraine patients have elevated oxidative stress. On the other hand, Geyik et al. study (19) has noted no statistically significant difference in total oxidant status, total antioxidant status, and oxidative stress index between migraine patients and controls; however, a significantly elevated plasma level of 8-hydroxy-2′-deoxyguanosine (8-OHdG) has been noted in migraine patients. Plasma 8-OHdG reflects oxidative damage induced by ROS to nuclear and mitochondrial DNA (20), also, reflects oxidative stress and mitochondrial dysfunction (21).

In addition, CSD, a hallmark of migraine pathogenesis, can cause oxidative stress (22). In addition, CSD is altered by pro-oxidant/antioxidant balance. Pro-oxidants potentiate and antioxidants prevent CSD (23). In fact, common triggers of migraine have the ability to generate oxidative stress; mechanisms include mitochondrial dysfunction, calcium excitotoxicity, activation of microglia, activation of NADPH oxidase, and as a byproduct of MAO (monoamine oxidase), cytochrome P450, or NO synthase (24). Collectively, it is indicated that oxidative stress is an important hallmark of migraine disease.

On a molecular basis, the TRPA1 (Transient receptor potential ankyrin subtype 1) ion channels in nociceptors allow the release of calcitonin gene-related peptide (CGRP) from dural afferents upon activation, mediating neurogenic inflammation, and migraine behavioral picture in animal models (25). Indeed, oxidative stress is an activator of the TRPA1 channel (25). Consequently, the TRPA1 receives elevated oxidative stress and initiate a neurogenic inflammatory response in migraine disease. In other words, TRPA1 is the bridge between oxidative stress and neuroinflammation in migraine.

### Neuroinflammation

Neurogenic inflammation describes the phenomenon of arteriolar vasodilation, plasma protein extravasation, and degranulation of mast cells, caused by the release of potent vasoactive neuropeptides (mainly CGRP, substance P, and neurokinin A) from activated peripheral nociceptive nerve terminals. In fact, the involvement of theses neuropeptides in migraine disease is evident (26). To emphasize, in Cui et al. study (27), microglial cells were activated significantly in response to CSD quantified by ^11^C-PK11195 PET, indicating a neuroinflammatory process mediating one hallmark (CSD) of migraine disease. In addition, in Karabulut et al. study (28), neutrophil/lymphocyte ratio (NLR) was elevated during a migraine attack. NLR produced from circulating neutrophils and lymphocytes counts, is considered an important marker assessing systemic inflammation (29). Collectively, it is indicated that neuroinflammation is an important hallmark of migraine disease.

### Mitochondrial Dysfunction

Mitochondrial dysfunction produces high levels of ROS favoring oxidative stress (30). Also, it impairs the cellular aerobic respiratory capacity predisposing to CSD through neuronal and glial energy failures (30). In a rat model of migraine, trigeminal neurons have been shown to have abnormal mitochondrial biogenesis capacity represented by the decreased mitochondrial DNA number of copies as well as the altered mRNA levels of peroxisome proliferator-activated receptor-gamma coactivator-1a (31), an essential regulatory factor in mitochondrial biogenesis (32). In addition, mitochondrial morphologic abnormalities have been found in migraine disease (30). To add, up to date, two polymorphisms in mitochondrial DNA have been associated with migraine susceptibility (30). Collectively, it is indicated that mitochondrial dysfunction is an important hallmark of migraine disease.

## Riboflavin Ameliorates Oxidative Stress, Mitochondrial Dysfunction, and Neuroinflammation

### Riboflavin Ameliorates Oxidative Stress

**Figure 1:**
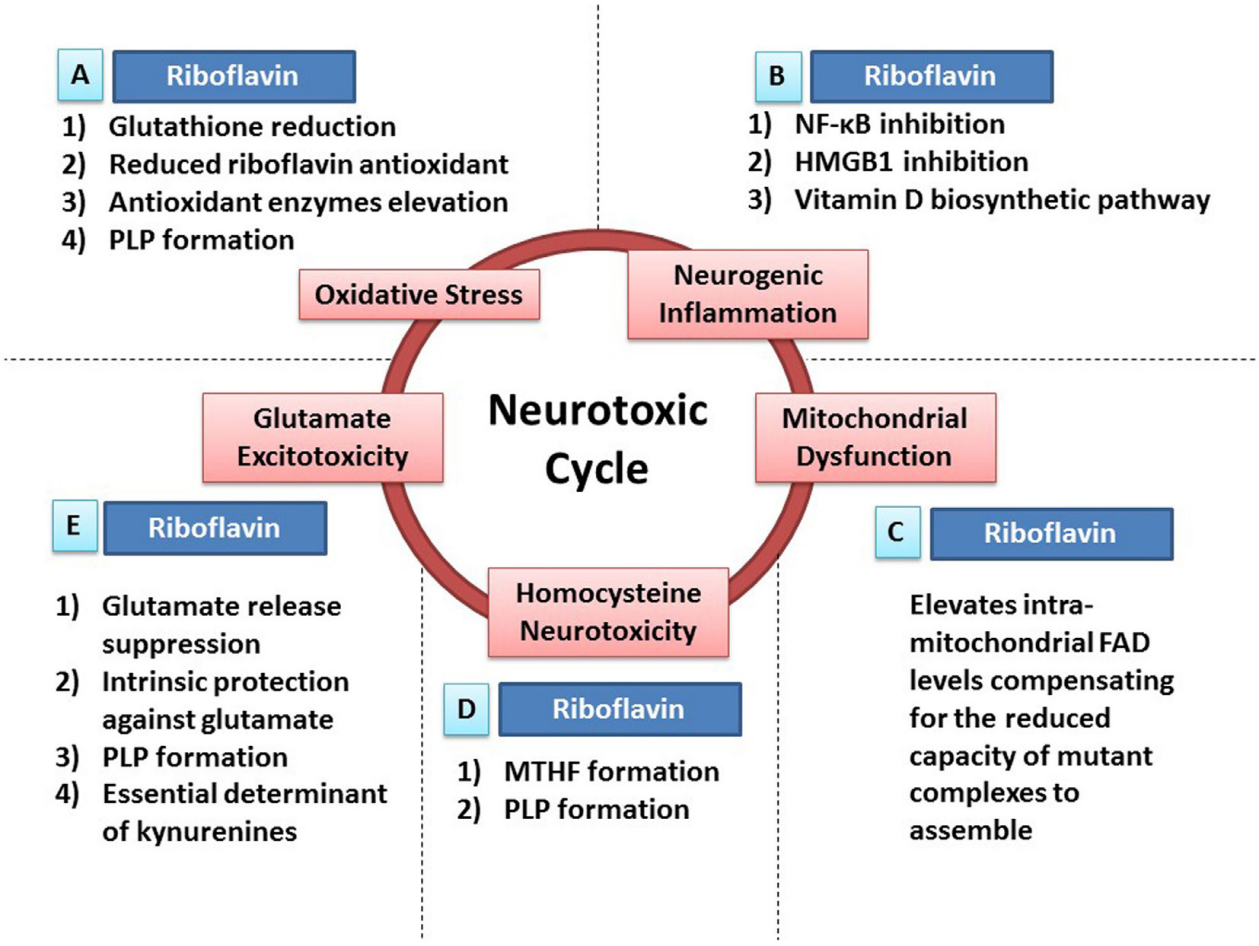
Riboflavin protects against neurotoxicity through ameliorating oxidative stress, mitochondrial dysfunction, neurogenic inflammation, glutamate excitotoxicity, and homocysteine neurotoxicity. Oxidative stress, mitochondrial dysfunction, neurogenic inflammation, glutamate excitotoxicity, and homocysteine neurotoxicity are involved in neurodegeneration and neurotoxicity. Also, those neurotoxic factors have the ability to cause each other leading to the formation of a neurotoxic cycle. Riboflavin is capable of attacking this proposed neurotoxic cycle *via* multiple neuroprotective mechanisms that tackle different neurotoxic factors in this neurotoxic cycle. **(A)** In fact, riboflavin attacks oxidative stress *via* its antioxidant potential. First, glutathione reductase requires riboflavin for its action to reduce oxidized glutathione increasing the levels of reduced (active) glutathione. Second, riboflavin has independent antioxidant action through its reduced form (dihydroriboflavin). Third, riboflavin has the ability to elevate antioxidant enzymes levels such as SOD and catalase. Fourth, riboflavin is required for the formation of pyridoxal phosphate (PLP), the active vitamin B6, which has its own antioxidant activity (see Riboflavin Is Required for the Formation of Pyridoxal Phosphate). **(B)** In addition, riboflavin attacks neurogenic inflammation either directly or indirectly. Riboflavin has the ability to inhibit NF-κB and high-mobility group protein B1 (HMGB1), nuclear factors involved in inflammatory processes, demonstrating its direct anti-inflammatory activity. On the other hand, multiple enzymes in the biosynthetic pathway of vitamin D are riboflavin-dependent enzymes, thus, riboflavin exerts its indirect anti-inflammatory activity *via* its essential role in vitamin D synthesis, which has a potent anti-inflammatory activity. **(C)** Furthermore, administration of riboflavin is capable of elevating the intra-mitochondrial levels of flavin adenine dinucleotide (FAD), which will compensate for the reduced capacity of dysfunctional complexes to assemble. As a result, riboflavin aims to normalize mitochondrial function in dysfunctional states. **(D)** Moreover, elevated homocysteine levels exhibit neurotoxic effects. Riboflavin-dependent enzymes are critical steps in the synthesis of methyltetrahydrofolate (MTHF) and PLP. MTHF and PLP are required for the actions of homocysteine metabolizing enzymes; methionine synthase and cystathionine b-synthase, respectively (see Riboflavin Is Required for Homocysteine Metabolism). **(E)** Additionally, riboflavin has the ability to attack glutamate excitotoxicity. In fact, riboflavin inhibits the endogenous neuronal release of glutamate reducing its excitotoxicity potential. In addition, both riboflavin and PLP (riboflavin is required for its synthesis) have their intrinsic protective properties against glutamate toxicity by increasing the survival of neurons exposed to glutamate toxicity after being treated with riboflavin or PLP. Also, both riboflavin and PLP are essential determinants of the tryptophan–kynurenine pathway, which produce neuroactive compounds known as kynurenines that influences glutamate receptors, hence, modulating glutamate excitotoxicity potential (see Riboflavin as a Determinant of the Kynurenine Pathway and Riboflavin Can Ameliorate Glutamate Toxicity; Which Is Implicated in Parkinson’s Disease and Migraine).

One of the underestimated antioxidants is riboflavin (Figure 1). In fact, there is a significant inverse linear correlation between riboflavin intake and MDA, a lipid peroxide, as found in a Moscow-based cross-sectional study (33); emphasizing the protective ability of riboflavin against lipid peroxidation, and consequently against oxidative stress. Indeed, multiple animal studies have shown that riboflavin-deficient states induce an elevation in lipid peroxidation markers, as well as, administration of riboflavin could induce reductions in those markers (33). To elaborate, riboflavin antioxidant function could be attributed to the glutathione redox cycle, the reduction-oxidation reactions of riboflavin itself, and the riboflavin effects on antioxidant enzymes activities.

First, glutathione is a major endogenous antioxidant against oxidative stress and lipid peroxidation. Reduced glutathione, the active form of this antioxidant, becomes oxidized, deactivated, during its antioxidant activity, thus, requiring reduction through glutathione reductase to regain its antioxidant activity. This enzyme requires the FAD coenzyme form of riboflavin for this reduction reaction, thus, emphasizing the role of riboflavin in the formation of reduced, active, glutathione (34). In fact, several animal studies have shown a decrease in reduced glutathione levels following a decrease in riboflavin intake. On the other hand, human erythrocyte glutathione levels were not significantly different in comparison between riboflavin-deficient humans and normal subjects as shown in one study, which comes in line with some animal studies results as well. This no difference state could be attributed to a compensatory increase in glutathione endogenous biosynthesis, or glutathione reductase ability to continue its normal activity despite low riboflavin states. However, when those glutathione normal riboflavin-deficient subjects face an oxidative challenge, such as ethanol intake, their glutathione levels decrease significantly in comparison with normal riboflavin subjects, as shown in one study (33).

Second, it has been shown that the oxidation of dihydroriboflavin, the reduced riboflavin, forming oxidized riboflavin can deactivate lipid peroxides, emphasizing the independent antioxidant property of riboflavin (33). Also, riboflavin has been suggested to have a direct activity against mutagen produced free radicals (35). Indeed, the protection of hepatocytes against reperfusion injury, in a state of ischemic liver, has been attributed to the independent antioxidant property of riboflavin (36).

Third, riboflavin effects on antioxidant enzymes activities, including superoxide dismutase (SOD) (34), glutathione peroxidase and catalase, have been reported with controversial results. It has been shown that riboflavin therapy can elevate cardiomyocytes SOD activity in diabetic cardiomyopathy state (33). Also, in a 2016 published study, Yu et al. has stated that riboflavin therapy can prevent abdominal aortic aneurysm through the endogenous activation of SOD in aneurysm walls, decreasing ROS levels (37). In another study, SOD and catalase activities have been significantly reduced by riboflavin-deficient diet for 12 weeks in fish (33).

Important to note, UV-B irradiation, the atmospherically predominant UV radiation, has been shown to reduce the neuroprotective effects of riboflavin both *in vitro* and *in vivo*. It has been suggested that riboflavin therapy increases miR-203 expression, which inhibits c-Jun expression, thus increasing neuronal survival (38). UV-B irradiation can modulate this signaling pathway, as well as, can induce photodegradation in this photosensitive vitamin (38). In fact, riboflavin-excess diet combined with light exposure can induce reduction in retinal photoreceptor layer through oxidants production (39). Indeed, those riboflavin-UV-B interaction generated oxidants can destroy DNA and RNA bases; however, antioxidants such as ascorbic acid provide protection against this photodegradation (40). Sunlight has been suggested to have similar DNA and RNA photodegradative effects in presence of riboflavin (41).

### Riboflavin Ameliorates Mitochondrial Dysfunction

Case reports of mitochondrial diseases have emphasized the beneficial effects of riboflavin administration. In two patients with complex I deficiency associated myopathy, complex I activity has been normalized upon riboflavin therapy with an evident clinical improvement (42). In comparison with controls, complex I activity has been increased from 16 to 47% upon high dose riboflavin therapy (43). Genetic testing has shown that *ACAD9* is involved in complex I function, deficiency state, and responsiveness to riboflavin (44–46). Continuous clinical response to riboflavin therapy for a 3-year period has been reported in a female patient with skeletal myopathy attributed to complex I deficiency (47). In addition to complex I deficiency patients, riboflavin administration has been used in complex II deficiency patients, resulting in moderate clinical improvement, stable clinical picture, and prevented disease progression, as well as a twofold elevation in complex II activity *in vitro* (48). In complex IV deficiency, which presumably is destabilized by complex I deficiency, riboflavin has been shown to improve its activity (49). Riboflavin administration elevates the intra-mitochondrial FAD levels, compensating for the reduced capacity of mutant complexes to assemble (44). In electron-transport flavoprotein dehydrogenase (*ETFDH*) gene mutations, riboflavin therapy can lead to clinical improvements (50).

### Riboflavin Ameliorates Neuroinflammation

Riboflavin has the ability to suppress nuclear factor-kappaB (NF-κB) activity exerting an anti-inflammatory property. In fact, it can inhibit chymotrypsin-like and trypsin-like proteasomal activities. This proteasomal inhibiting role can lead to a reduced proteasomal elimination of ubiquinated P-Iκ (phosphorylated-inhibitor kappa), inhibiting nuclear translocation of NF-κ, suppressing NF-κB activation and its consequences of tumor necrosis factor alpha (TNF-α), and NO production (34, 51, 52). In a PD model, a reduction in microglial activation has been demonstrated by doxycycline through the suppression of NF-κB nuclear translocation (53). Also, through NF-κB inhibition, prophylactic α-asarone suppresses MPTP (1-methyl-4-phenyl-1,2,3,6-tetrahydropyridine) microglial activation and parkinsonian behavioral deficits (54). In addition, chronic inflammation can cause CNS pain conditions; suppressing astroglial NF-κB can reduce CNS inflammatory pain (55). Prophylactic valproate suppresses NF-κB activation in the trigeminocervical complex (56) as well as, prophylactic atorvastatin suppresses NF-κB activation in trigeminal nucleus caudalis (57); both alleviate nitroglycerin-induced migraine, indicating a vital role of NF-κB activation in migraine pathogenesis (56–58). Indeed, NF-κB has an essential role in neuroinflammation (59).

In addition to inhibiting NF-κB, riboflavin inhibits the release and expression of High-mobility group protein B1, a nuclear factor involved in sepsis regulation and other immune-mediated conditions with a critical role in sepsis-associated multiple organ failure (60). In a staphylococcal infection setting, riboflavin has demonstrated its anti-inflammatory property through reductions in NF-κB, COX2 (cyclooxygenase 2), TNF-α, NO, IL-1β (interleukin 1 beta) reducing iNOS (inducible NO synthase) synthesis, as well as an elevation in anti-inflammatory cytokine IL-10 (interleukin 10), and modulation of MCP-1 (monocyte chemoattractant protein 1) function, a potent chemoattractant (34).

On the other hand, riboflavin has an indirect anti-inflammatory activity through vitamin D metabolism. In fact, essential enzymes in the biosynthetic pathway of vitamin D are based on flavins, including flavin-dependent monooxygenases and oxidoreductases (61). Indeed, in an animal study, riboflavin deficiency has resulted in a significant reduction in serum 25(OH)D with moderate reduction in serum calcium; both alleviated with vitamin D administration (61). It has been shown that vitamin D3 administration suppresses microglial activation in a lipopolysaccharide-activation model (62). Indeed, prophylactic vitamin D3 has improved dopaminergic neuronal survival significantly in an MPTP model of PD through suppression of microglial activation (63). As suggested, vitamin D3 elevates microglial IL-10 expression; inducing suppressor of cytokine signaling-3, leading to a decline in pro-inflammatory cytokines expression in microglia (64).

## Riboflavin is Required for the Formation of Pyridoxal Phosphate (PLP)

**Figure 2:**
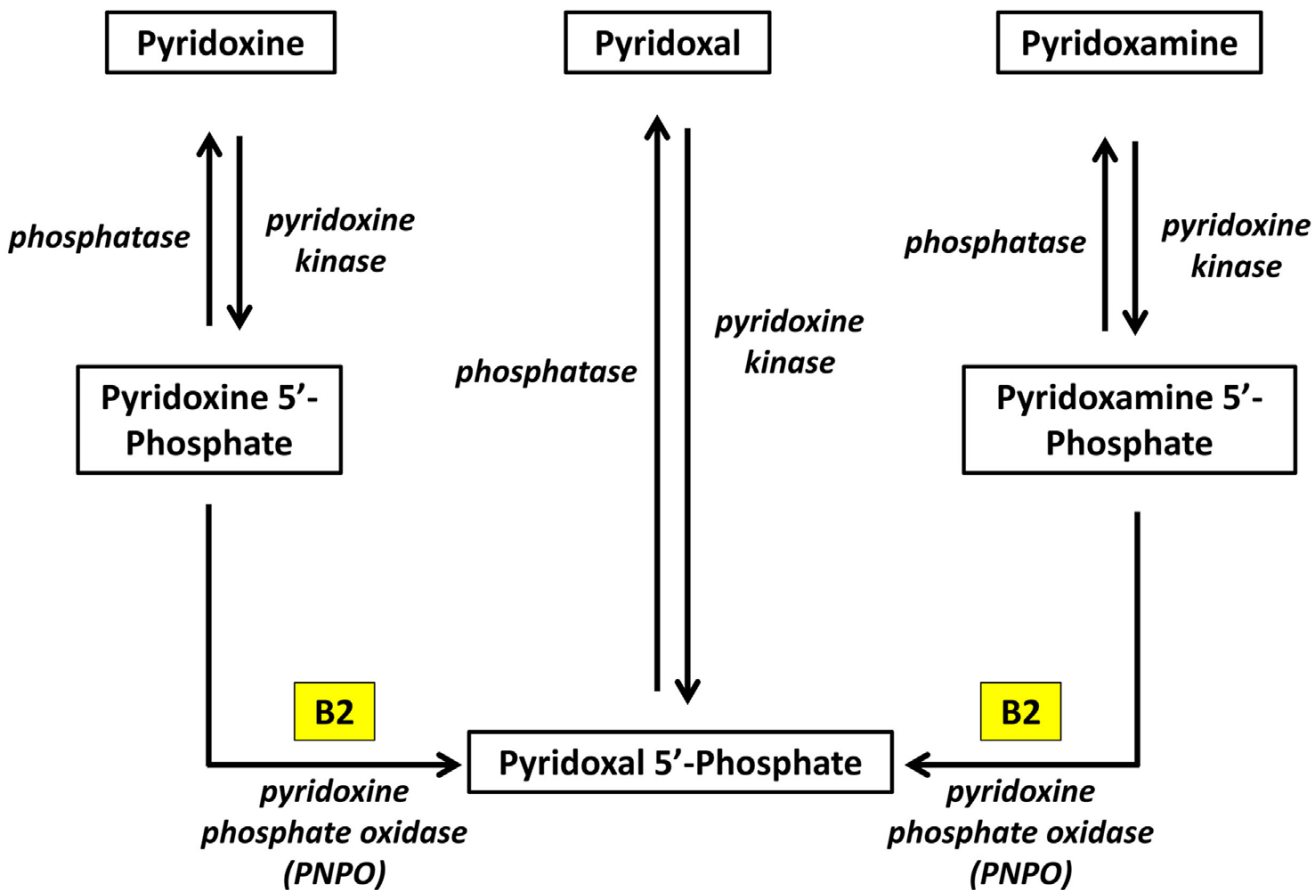
Riboflavin is required for the formation of Pyridoxal phosphate (68). Pyridoxine, pyridoxal, and pyridoxamine are forms of vitamin B6 (vitamin B6 vitamers). Through pyridoxine kinase, those vitamers will form pyridoxine 5′-phosphate, pyridoxal 5′-phosphate, pyridoxamine 5′-phosphate, respectively. These reactions are reversible with phosphatases. Pyridoxal 5′-phosphate (PLP) is the active form of vitamin B6. Consequently, pyridoxine 5′-phosphate and pyridoxamine 5′-phosphate must be converted to PLP. Pyridoxine phosphate oxidase (PNPO) is the enzyme required for this conversion and formation of the active PLP from pyridoxine and pyridoxamine. PNPO requires riboflavin (B2) as its main cofactor.

Pyridoxal phosphate is the active form of pyridoxine. Pyridoxine phosphate oxidase (PNPO) synthesizing PLP requires riboflavin as its main cofactor (65). In fact, conditioned pyridoxine deficiency can arise from riboflavin deficiency (66). Administration of low dose riboflavin to individuals with decreased EGRAC or PLP levels has significantly enhanced the status of the decreased nutrient whether its riboflavin or PLP (67). Consequently, riboflavin is considered the limiting nutrient (67) (Figure 2).

Regarding PD, a Japanese case–control study has shown that low intake of vitamin B6 was associated with an increased risk of PD (69). In addition, a Netherlands based cohort study and a Germany-based case–control study have found that a decreasing risk of PD is associated with a high intake of vitamin B6 (69). Collectively, vitamin B6 has a neuroprotective property manifested by its essential role in dopamine biosynthesis, as well as, its independent antioxidant ability (69). Indeed, in 1941, vitamin B6 supplementation has improved parkinsonian behavioral deficits in a subgroup of PD patients (70). In fact, PLP insufficient intracellular stores have been correlated with PD (70). As been asserted, carbidopa causes irreversible binding of PLP and PLP-dependent enzymes depleting PLP bodily stores (70, 71). Consequently, carbidopa administration has been associated with PD elevated death rate, progressive neurodegenerative course, and l-DOPA tachyphylaxis (70).

Regarding migraine headache, administration of pyridoxine has decreased headache attack severity and duration in comparison with placebo, with no effect on frequency (72). It has been reported that 1–month period administration of 150 mg pyridoxine resulted in a significantly reduced headache attack severity. Also, a significant reduction in migraine headache attack severity, frequency, and disability using a combination of pyridoxine, folate, and cobalamin has been reported (72).

## Riboflavin as a Determinant of the Kynurenine Pathway

**Figure 3:**
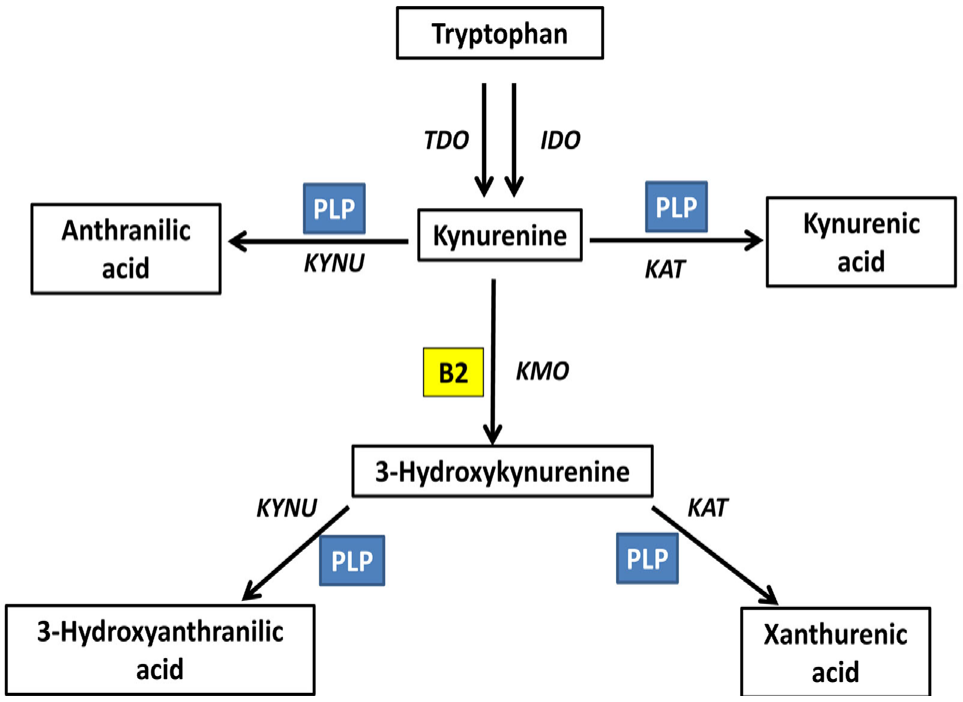
Riboflavin is an essential determinant of the kynurenine pathway (73). Kynurenine pathway is the main tryptophan catabolism pathway, with neuroactive metabolites known as kynurenines. This pathway is determined by vitamin B2 status, indicated by plasma riboflavin, and B6 status, indicated by circulating PLP; since both vitamins are essential cofactors. Flavin adenine dinucleotide is required for the formation of 3-hydroxykynurenine. PLP is required for the formation of anthranilic acid, 3-hydroxyanthranilic acid, kynurenic acid, and xanthurenic acid. TDO, tryptophan 2,3-dioxygenase; IDO, indoleamine 2,3-dioxygenase; KAT, kynurenine aminotransferase; KYNU, kynureninase; KMO, kynurenine 3-monooxygenase; PLP, pyridoxal phosphate; B2, riboflavin.

Kynurenine pathway is the main tryptophan catabolism pathway, with neuroactive metabolites known as kynurenines. This pathway is determined by vitamin B2 status, indicated by plasma riboflavin, and B6 status, indicated by circulating PLP; since both vitamins are essential cofactors. FAD is required for the formation of 3-hydroxykynurenine. PLP is required for the formation of anthranilic acid, 3-hydroxyanthranilic acid, kynurenic acid, and xanthurenic acid. Plasma riboflavin, as well as riboflavin intake, is positively associated with 3-hydroxyanthranilic acid and xanthurenic acid (73). PLP is positively associated with kynurenic acid, 3-hydroxyanthranilic acid and xanthurenic acid, all formed by PLP-based enzymes, and negatively associated with 3-hydroxykynurenine, metabolized by PLP-based enzymes; however, vitamin B6 intake showed null associations with kynurenines (73). Xanthurenic acid is determined by both riboflavin and PLP interactively (73) (Figure 3).

Glutamate receptors, including NMDA and metabotropic receptors, are influenced by the kynurenines. In fact, kynurenic acid is an antagonist of NMDA and all ionotropic glutamate receptors (74, 75). Xanthurenic acid is an activator of type-2 metabotropic glutamate receptors (74). Picolinic acid is neuroprotective, considered the brain main metal chelator (75). However, quinolinic acid is an agonist of NMDA (74, 75). 3-hydroxykynurenine, 3-hydroxyanthranilic acid, and 5-hydroxyanthranilic acid are neurotoxic with ROS generation properties (75).

Important to know, kynurenine pathway abnormalities have been reported in migraine (74) and PD (76). Chronic migraine patients have shown astonishing elevation of anthranilic acid as well as xanthurenic acid, to a moderate extent, with a decline in all other kynurenines (74). PD studies have shown a decline in kynurenic acid levels with elevation in 3-hydroxykynurenine levels favoring neurotoxic states in the frontal cortex, putamen and substantia nigra pars compacta brain regions (75, 77). In addition, an imbalance between the decreased astroglial kynurenic acid and the increased microglial quinolinic acid has been shown in PD (75). In fact, MPTP depletes kynurenic acid level (77). Improving kynurenic acid levels could be an effective neuroprotective strategy in PD, migraine, and neurodegenerative diseases (78).

## Riboflavin Can Ameliorate Glutamate Toxicity; Which is Implicated in PD and Migraine

Reactive oxidant species may contribute to glutamate excitotoxicity states *via* suppression of astroglial glutamate transporters and glutamine synthase (24), increasing glutamate/glutamine levels and cortical calcium levels (79). In addition, mitochondrial dysfunction up-regulates NMDA glutamate receptors (80). Also, glutamate excitotoxicity, itself, forms fragmented mitochondria, contributing to oxidative stress through the dysfunctional mitochondria (80). Collectively, oxidative stress, mitochondrial dysfunction, and glutamate excitotoxicity are causing each other forming a neurotoxic vicious cycle (80). Upregulation of SOD2 and antagonism of NMDA receptors can manipulate this vicious cycle (80).

As a matter of fact, riboflavin has the potential to manipulate this neurotoxic vicious cycle of oxidative stress, mitochondrial dysfunction, and glutamate excitotoxicity. As shown in Section “Riboflavin Ameliorates Oxidative Stress, Mitochondrial Dysfunction, and Neuroinflammation,” riboflavin has antioxidant properties and upregulates antioxidant enzymes especially SOD, also, supplementation with riboflavin will elevate the intra-mitochondrial FAD levels compensating for the decline in the capacity to assemble of dysfunctional mitochondrial complexes. Indeed, riboflavin suppresses the cortical neuronal endogenous release of glutamate *via* reduction in the activity of presynaptic voltage-gated calcium channels, inhibiting the exocytosis of glutamate vesicles (81). Thus, riboflavin protects against glutamate excitotoxicity by decreasing glutamate release in the first place, decreasing its concentration in the synapses, and subsequently its excitotoxicity potential. In addition, riboflavin and pyridoxine, which requires riboflavin for its activation, have been shown to have intrinsic neuroprotective properties against glutamate excitotoxicity; as shown in experimental studies using cerebellar granular cell cultures exposed to glutamate after being treated with riboflavin or pyridoxine (82, 83). In addition, both riboflavin and PLP are essential determinants of the kynurenine pathway which influence glutamate receptors, hence, glutamate excitotoxicity potential, as shown in Section “Riboflavin as a Determinant of the Kynurenine Pathway.”

Concerning PD, glutamate excitotoxicity contributes to dopaminergic neuronal loss (84); however, it is unlikely to be a sole action of glutamate excitotoxicity (85). Indeed, glutamate excitotoxicity contributes to elevated intra-neuronal calcium levels influencing neuronal survival. As a matter of fact, intra-neuronal calcium levels, regulated by mitochondria and NMDA activity, are essential in maintaining neuronal survival. Indeed, intra-neuronal calcium overload due to an imbalance between NMDA calcium-increasing and mitochondrial calcium-lowering activities may trigger cellular death (85); through activation of cellular phospholipases, endonucleases, and proteases degrading intracellular structures (85). Also, glutamate excitotoxicity NMDA-mediated intra-neuronal calcium overload elevates NO synthesis contributing to oxidative stress particularly the production of reactive nitrogen species which are able to cause defects in DNA and protein phosphorylation pathway (85). In addition, glutamate signal transduction in neurons is augmented by dopamine, therefore, nigral neurons, i.e., substantia nigra pars compacta neurons, are highly susceptible to glutamate excitotoxicity effects (85). Even under normal levels of glutamate, mitochondrial dysfunction will increase the susceptibility of nigral neurons to glutamate excitotoxicity effects (85). On the other hand, *parkin* gene encodes an E3 ubiquitin ligase involved in ubiquitin-proteasome pathway. As mutated, it causes an early onset autosomal recessive PD. As a matter of fact, parkin has been involved in supporting mitochondrial normal structure as well as maintaining stable glutamatergic synapses (85). Once mutated, outgrowth of glutamatergic synapses has been noted (85). Therefore, mutations in *parkin* gene increase the susceptibility to glutamate neurotoxicity predisposing to the early onset neurodegeneration of PD. This emphasizes the role of glutamate excitotoxicity in the pathogenesis of PD.

Concerning migraine, glutamate has been suggested to have a role in its pathogenesis (86). As a matter of fact, NMDA antagonists may decrease trigeminovascular pain signal transmission *in vivo* (15). Indeed, the antiepileptic drug, topiramate, has been used in migraine prophylaxis successfully (87). Topiramate blocks the ionotropic glutamate receptors in the trigeminothalamic pathway, as well as sodium and calcium channels with GABA signal enhancement (87); emphasizing the role of glutamate excitotoxicity in migraine pathogenesis.

## Riboflavin is Required for Homocysteine Metabolism

**Figure 4:**
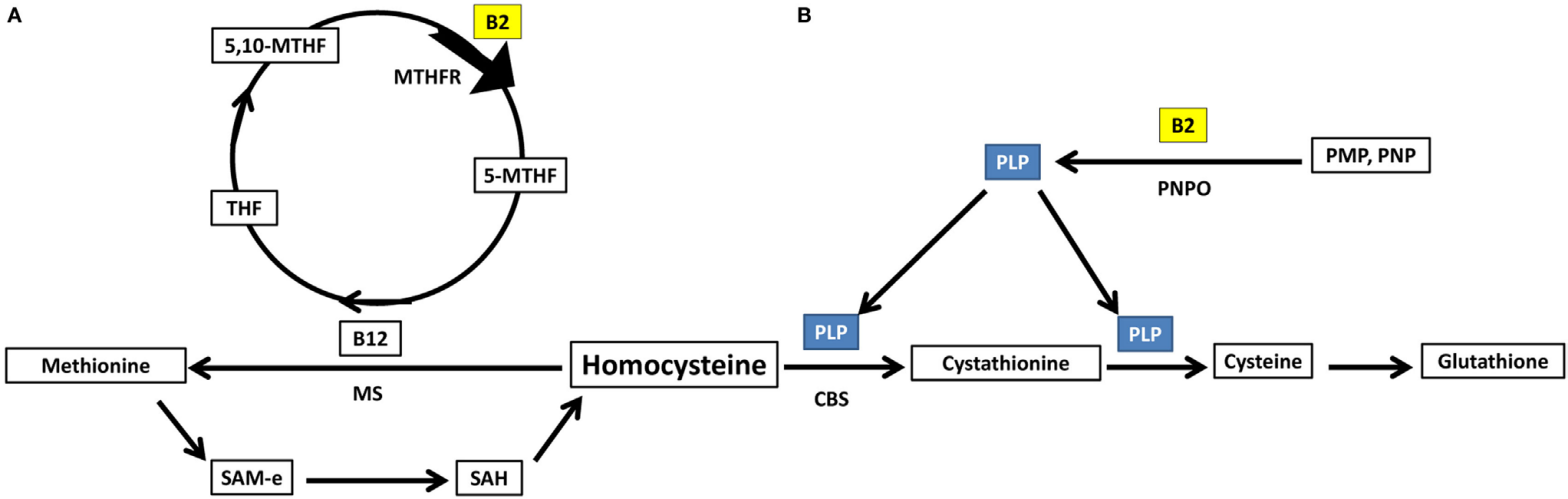
Riboflavin has essential role in homocysteine metabolic pathways of re-methylation and transsulfuration. **(A)** Homocysteine undergoes re-methylation forming methionine through MS which requires methylated b12. The methyl group is donated from 5-methyltetrahydrofolate, synthesized *via* action of the riboflavin-dependent enzyme MTHFR on 5,10-methylenetetrahydrofolate. **(B)** The second fate of homocysteine is to undergo transsulfuration through CBS forming cystathionine and glutathione. This pathway requires PLP as a cofactor. PLP requires riboflavin for its synthesis from vitamin B6 phosphorylated vitamers. THF, tetrahydrofolate; 5,10-MTHF, 5,10-methenyltetrahydrofolate; MTHFR, methylenetetrahydrofolate reductase; 5-MTHF, 5-methyltetrahydrofolate; B12, cobalamin; MS, methionine synthase; SAM-e, S-adenosyl methionine; SAH, S-adenosylhomocysteine; PLP, pyridoxal phosphate; CBS, cystathionine b-synthase; PNPO, pyridoxine/pyridoxamine phosphate oxidase; PMP, pyridoxamine phosphate; PNP, pyridoxine phosphate.

Homocysteine can either be acted upon by cystathionine b-synthase forming cystathionine and glutathione (88), requiring PLP as a cofactor, or methionine synthase forming methionine (88), requiring methyl B12 as a cofactor and 5-methyltetrahydrofolate as a methyl donor. Important to note, both PLP and 5-methyltetrahydrofolate require riboflavin in their biosynthesis using the active forms of riboflavin, FMN, and FAD, respectively (89) (Figure 4).

Indeed, requiring FAD as coenzyme, methylenetetrahydrofolate reductase (MTHFR) is responsible for the formation of 5-methyltetrahydrofolate. *MTHFR* gene C677T polymorphism increases the genetic predisposition to hyperhomocysteinemia. Homozygous individuals with this polymorphism (TT genotype) have a 12% prevalence (89). Indeed, TT genotype individuals have a decline in their MTHFR activity; attributed to its FAD cofactor loss (90). Riboflavin administration has been shown to be effective against TT genotype-associated hyperhomocysteinemia (89) and confirmed to be an independent determinant of homocysteine levels in those individuals (90). Moreover, it has been shown that riboflavin has a negative association with homocysteine levels in normal MTHFR activity individuals, CC genotype; emphasizing the unrestricted effect of riboflavin on homocysteine metabolism despite *MTHFR* genotypes (91). On the other hand, lowering homocysteine with folate supplementation could deplete riboflavin stores; requiring the additional riboflavin supplementation to maximize MTHFR activity and save vitamin stores (91).

As a matter of fact, *MTHFR* C677T polymorphism has been shown to increase risk of PD in Europeans (92, 93), and Asians (93) with conflicting data (92), and has been shown to significantly increase risk of Migraine in Asians (94). Furthermore, elevated CSF homocysteine level has been associated with migraine (95), migraine aura (95), PD (96), and post l-DOPA therapy (97) attributed to S-adenosylmethionine consumption and consequent S-adenosylhomocysteine elevation (88). In fact, the elevated homocysteine and reduced S-adenosylmethionine in PD has been ascribed to physiologic aging associated reduction in homocysteine metabolism cofactors (88). In addition, PD hyperhomocysteinemia has been linked to dementia, depression, disease progression, cognitive deterioration, and vascular diseases. Indeed, homocysteine neurotoxic properties have been attributed to NMDA receptor stimulation, auto-oxidation elevating oxidative stress, mitochondrial complex I inhibition, and allosteric D2 receptor antagonism (88).

## Riboflavin Administration in PD and Migraine

Up to our knowledge, there is only one clinical trial that studied the effects of high dose riboflavin on PD patients. This study used an oral dose of 30 mg riboflavin every 8 h, in combination with the usual symptomatic treatment of PD, for a 6-month period in 19 patients with PD and low-riboflavin status despite normal general nutritional status (98). Since PD patients have higher consumption of red meat in comparison with healthy controls, dietary red meat was eliminated during this 6-month period study (98). Enhanced motor capacity was noted in all subjects in a progressive manner that reaches a plateau during the first 3 months of the study; while in 5 out of the 19 subjects, motor capacity continues to recover in every month in the 6-month period (98). In this study, motor capacity enhanced from 44 to 71% in average (98). 100% motor capacity has been reached by three patients within the first 3 months of this study (98). However, a consideration was raised by a commentary on this paper (99). Ferraz et al. has pointed to the effects of low-protein diet on enhanced levodopa absorption (99). According to the commentary, the improved motor capacity in those patients could be the result of the enhanced absorption of levodopa rather than the administration of riboflavin (99).

Concerning riboflavin therapy in migraine patients, a recent systematic review has tackled this issue (100). Riboflavin role in migraine therapy is prophylactic, in other words, it affects migraine attacks frequency, severity, duration, and related disability, and facilitates acute therapies of migraine attacks (100). According to the American Academy of Neurology, riboflavin is considered a level B therapy in migraine prophylaxis (100). Riboflavin, as a migraine prophylactic agent, has been studied in adults and children. Five clinical trials involving adult patients have been evaluated in a systematic review with positive results (100). Indeed, a significant reduction in migraine attack frequency, 59% reduction, has been noted in a randomized double-blind placebo-controlled trial using 400 mg/day of riboflavin (100). Also, two open-label trials using 400 mg/day of riboflavin have decreased migraine attack frequency from 4 attacks per month to 2 attacks per month, and from 8.7 attacks per month to 2.9 attacks per month, respectively (100). In addition, as migraine prophylactic agents, 400 mg/day of riboflavin was comparable with 500 mg/day of sodium valproate and 100 mg/day of riboflavin was comparable with 80 mg/day of propranolol, with no statistical differences between groups in their prophylactic actions, with a more favorable side effect profile in the riboflavin groups (100). On the other hand, clinical trials involving children and adolescent patients have mixed results (100). Two randomized double-blind placebo-controlled trials using 50–200 mg/day of riboflavin had failed to produce any significant benefit in migraine prophylaxis involving 5- to 15-year-old patients (100). However, a randomized double-blind placebo-controlled trials using 400 mg/day of riboflavin in adolescent patients between 12- and 19-year-old has demonstrated significant positive results in reductions of migraine attack frequency and related disability (100).

## Conclusion

Riboflavin is a potential neuroprotective agent. In fact, riboflavin has demonstrated its ability to tackle significant pathogenesis-related mechanisms in neurological disorders, exemplified by the ones attributed to the pathogenesis of PD and migraine. Indeed, riboflavin ameliorates oxidative stress, mitochondrial dysfunction, neuroinflammation, and glutamate excitotoxicity; all of which are involved in the pathogenesis of a wide range of neurological disorders. In addition, riboflavin is required for pyridoxine activation. Riboflavin and PLP, the active form of pyridoxine, play essential roles in homocysteine metabolism, and tryptophan-kynurenine pathway. Indeed, any accumulation of homocysteine or kynurenines due to vitamin insufficiency can lead to significant neurological consequences. Taking into consideration the limited riboflavin absorption and utilization in 10–15% of global population, long term riboflavin insufficiency could participate in the development of multiple neurological disorders, emphasizing the importance of long-term riboflavin-sufficient diet especially in vulnerable populations. Indeed, randomized double-blind placebo-controlled trials, with extended time frame and large number of patients, are encouraged to clinically evaluate the role of riboflavin in PD and migraine headache patients.

## Author Contributions

Both authors EM and SB have participated equally in article conception and design, acquisition of data, analysis and interpretation of data, drafting of manuscript, critical revision, and final approval of the version to be published. Also, both authors agree to be accountable for all aspects of the work, and ensure that accuracy and integrity questions of any part of the work are appropriately investigated and resolved.

## Conflict of Interest Statement

The authors declare that the research was conducted in the absence of any commercial or financial relationships that could be construed as a potential conflict of interest.
